# Antibiotic Use in Low and Middle-Income Countries and the Challenges of Antimicrobial Resistance in Surgery

**DOI:** 10.3390/antibiotics9080497

**Published:** 2020-08-09

**Authors:** Massimo Sartelli, Timothy C. Hardcastle, Fausto Catena, Alain Chichom-Mefire, Federico Coccolini, Sameer Dhingra, Mainul Haque, Adrien Hodonou, Katia Iskandar, Francesco M. Labricciosa, Cristina Marmorale, Ibrahima Sall, Leonardo Pagani

**Affiliations:** 1 Department of Surgery, Macerata Hospital, 62100 Macerata, Italy; 2 Trauma Service, Inkosi Albert Luthuli Central Hospital and Department of Surgery, Nelson R. Mandela School of Clinical Medicine, Durban 4058, South Africa; hardcastle@ukzn.ac.za; 3 Department of Emergency Surgery, Parma Maggiore Hospital, 43126 Parma, Italy; faustocatena@gmail.com; 4 Department of Surgery and Obs/Gyn, Faculty of Health Sciences University of Buea, Buea P.O. Box 63, South West Province, Cameroon; alainchichom@yahoo.com; 5 General, Emergency and Trauma Surgery Department, Cisanello University Hospital, 56100 Pisa, Italy; federico.coccolini@gmail.com; 6 School of Pharmacy, Faculty of Medical Sciences, The University of the West Indies, St. Augustine, Eric Williams Medical Sciences Complex, Uriah Butler Highway, Champ Fleurs 33178, Trinidad and Tobago; sameer.dhingra@sta.uwi.edu; 7 Unit of Pharmacology, Faculty of Medicine and Defence Health, Universiti Pertahanan Nasional Malaysia (National Defence University of Malaysia), Kuala Lumpur 57000, Malaysia; runurono@gmail.com; 8 Department of General Surgery, Regional Hospital Borgou, Faculty of Medicine, University of Parakou, Parakou P.O. Box 123, Benin; hodasm98@gmail.com; 9 Department of Pharmacy, Lebanese, International University, Beirut 1106, Lebanon; katia_iskandar@hotmail.com; 10 Global Alliance for Infections in Surgery, 4400-001 Vila Nova de Gaia, Portugal; labricciosafrancesco@gmail.com; 11 Department of Surgery, Università Politecnica delle Marche, 60126 Ancona, Italy; c.marmorale@univpm.it; 12 General Surgery Department, Military Teaching Hospital, Dakar 3006, Senegal; sall_i17@yahoo.fr; 13 Infectious Diseases Unit, Bolzano Central Hospital, 39100 Bolzano, Italy; lpagani.id@gmail.com

**Keywords:** antimicrobial resistance, antimicrobial prescription, infection control, surgical infections, low- and middle-income countries

## Abstract

Antimicrobial resistance (AMR) is a phenomenon resulting from the natural evolution of microbes. Nonetheless, human activities accelerate the pace at which microorganisms develop and spread resistance. AMR is a complex and multidimensional problem, threatening not only human and animal health, but also regional, national, and global security, and the economy. Inappropriate use of antibiotics, and poor infection prevention and control strategies are contributing to the emergence and dissemination of AMR. All healthcare providers play an important role in preventing the occurrence and spread of AMR. The organization of healthcare systems, availability of diagnostic testing and appropriate antibiotics, infection prevention and control practices, along with prescribing practices (such as over-the-counter availability of antibiotics) differs markedly between high-income countries and low and middle-income countries (LMICs). These differences may affect the implementation of antibiotic prescribing practices in these settings. The strategy to reduce the global burden of AMR includes, among other aspects, an in-depth modification of the use of existing and future antibiotics in all aspects of medical practice. The Global Alliance for Infections in Surgery has instituted an interdisciplinary working group including healthcare professionals from different countries with different backgrounds to assess the need for implementing education and increasing awareness about correct antibiotic prescribing practices across the surgical pathways. This article discusses aspects specific to LMICs, where pre-existing factors make surgeons’ compliance with best practices even more important.

## 1. A Global Alliance to Fight Antimicrobial Resistance across the Surgical Pathway

“A successful approach to infections should focus on collaboration among different healthcare professionals in order to share knowledge, and best practices”.

The Global Alliance for Infections in Surgery [1] instituted a multidisciplinary working group comprised of professionals from different countries, and with different backgrounds, in order to assess antibiotic prescription and use in low and middle-income countries (LMICs) with specific reference to the current global challenges of antimicrobial resistance (AMR) across the surgical pathway. The literature search for this narrative review was performed by searching computerized bibliographic databases, including Google Scholar and PubMed, using the following search terms: “antimicrobial resistance”, “antimicrobial prescription”, “low and middle-income countries”, and “surgical infections” with a 20-year retrospective cut-off. The exclusion criteria were articles without full text access, and non-English language. Additional references were identified from the cited references using hand-searching. No attempt was made to develop a systematic review and meta-analysis. The working group reviewed the original draft via email round-robin to critically review the draft and finalised the manuscript.

## 2. Antimicrobial Resistance: A Global Challenge

The discovery of penicillin by Alexander Fleming in the late 1920s has deeply changed medical practice. Antibiotics have saved millions of lives over years and have even been used prophylactically for the prevention of infectious diseases. However, we have now reached a crisis where many antibiotics are no longer effective. AMR is a natural phenomenon that happens as microorganisms evolve, however human activities have altered the pace at which microbes develop and spread resistance [2,3,4,5].

AMR is one of the major public health concerns of this century [6], threatening the practice of modern medicine, food security, and animal health. Although many factors can be linked to the phenomenon of AMR, a well-established relationship between antibiotic prescribing practices and use, and the emergence of AMR can be identified.

The drivers of AMR encompass not only antibiotic use, misuse, and abuse in human, animal, and environmental sectors, but also the dissemination of resistant microorganisms within and among these sectors, and therefore around the world [7,8]. Human, animal and environmental habitats are contiguous and interconnected, determining and contributing to the emergence and spread of AMR. The One Health approach focuses on this issue: the health of these habitats represents a risk to human health [9].

In May 2015, the World Health Assembly endorsed a global action plan to fight AMR. Five strategic objectives were set out: (1) strengthening both understanding and awareness of AMR; (2) improving knowledge through surveillance and research; (3) reducing the incidence of infection; (4) optimizing the use of antibiotic agents; (5) developing the economic case for sustainable investment, taking into consideration the needs of all countries, and increasing investment in new medicines, vaccines, diagnostic tools, and other interventions [10].

AMR is a complex, multifaceted problem that threatens human and animal health, the global economy, national and global security. In line with a One Health approach, healthcare plays an important role in preventing the emergence and spread of AMR. The success of modern medicine, including organ transplantation, cancer therapy, management of prematurity, or a surplus of advanced major surgeries, might not have been possible without effective antibiotics to control bacterial infections.

Patients often face multiple risk factors exposing them to AMR. Acute care facilities are incubators and highly susceptible vulnerable populations create an environment which facilitates both the emergence and transmission of resistant organisms, especially in intensive-care units managing surgical cases. Across the surgical pathway, surgical site infections (SSIs) remain a common postoperative complication worldwide despite the use of prophylactic antibiotics and other preventive measures [11,12], and represent an important burden mainly due to increasing AMR.

The problem of AMR seems to represent a special challenge in LMICs characterized by the scarcity of biological and epidemiological data, a lower level of awareness and the absence of locally developed guidelines in most sites. The few existing data are alarming. In a hospital based descriptive cross-sectional study conducted on 83 consented postoperative patients with clinical SSIs at Mbarara regional referral hospital in Uganda a very high AMR rate of bacteria isolated was reported [11]. Overall 86.0% of aerobic bacteria isolated were multidrug resistant (MDR). Importantly, 65.6% of Gram positive and 96.7% of Gram-negative isolates were MDR. Gram negative pathogens showed high resistance to ceftriaxone, sulfamethoxazole/trimethoprim, and gentamicin. With exception of *Enterococci* spp., all the isolated bacteria were resistant to ampicillin. All the isolated *Klebsiella* spp. were found to be MDR. *Staphylococcus aureus* isolates were all resistant to oxacillin. The isolation rate of MDR organisms was higher in emergency, males, and dirty wounds in relation to type of surgery, gender and class of surgical wound respectively. A study carried out at the university hospital of Parakou, Benin in 2017 revealed that 41.2% of the microbial germs isolated in the surgical departments during SSIs were MDR [13].

The appropriate use of antibiotics plays an essential role in optimizing clinical practice. Antibiotics may be lifesaving however these are often used inappropriately, especially when they are unnecessary, or when they are administered without taking into account pharmacokinetic principles, or when they are administered for an excessive duration [14]. The misuse and abuse of antibiotics is extensively recognized as a major driver of the selection of resistant microorganisms in patients, and for the continued development and dissemination of AMR globally. AMR knows no borders no gender and no race and has a well-known negative externality with frightening future prospects. It is not only an infectious disease problem but also a surgical problem threating to undermine all advances in the field [15].

Infections require not only prompt diagnosis and immediate source control, but also adequate antibiotic therapy [16]. Inappropriate antibiotic prescribing in surgery is widely reported, with patients at risk of receiving prolonged durations of antibiotics for prophylactic and therapeutic indications [17]. The compliance with antibiotic prescribing practices is important for all physicians all over the world but is even more important for physicians in LMICs where pre-existing factors make compliance with prescription practices even more difficult.

## 3. Antimicrobial Resistance in LMICs

AMR containment interventions in healthcare facilities have mostly been implemented in high-income countries. There is a persistent need to intervene in LMICs since the burden of AMR in LMICs is difficult to quantify, because surveillance activities to lead interventions require not only time, but also financial resources. Moreover, a basic background in epidemiology, microbiology, and communicable diseases should be required, in addition to expertise in data management and analysis [18,19]. In these countries, especially in rural hospitals and health facilities, routine microbiologic cultures and sensitivity testing may not be performed, due to a lack of workers, equipment, and financial resources. As a result, antibiotic therapy is empirical, and the few available antibiotics may be overused and misused. This approach appears to be relatively inexpensive, but may further increase the emergence and spread of AMR, and therefore sub-optimal clinical outcomes [20,21].

As far back as 2005 a publication from South Africa noted a rapid increase in the presence of resistant strains of infectious organisms across the levels of care with, as expected a higher rate among patients in tertiary facilities [22]. Recent publications from other African countries have shown similar concerning results: Work from Ghana shows resistant patterns are rife within a large teaching hospital, affecting particularly the extremes of age and surgical patients [23]. There is considerable variability in the access to and use of antibiotics across various LMICs with a pilot site review from India and Africa demonstrating that public sector facilities used older agents and private sector facilities more often accessed newer agents, while the usage appeared higher per-capita in India than South Africa, possibly due to more restrictive prescribing in the latter country or access to “over the counter purchase” in the former [24]. Antibiotic usage, examined in a utilisation review in Namibia, appears to be higher in females, in the large cities and the economically active age-groups (18–45 years), with broad-spectrum penicillins, cephalosporins and macrolides constituting over 80% of the usage, which potentially contributes strongly to resistance patterns [25]. In the context of this review the surgical services in many LMICs will be offered primarily in the large cities and this may reflect part of the problem of AMR in the surgical population.

In order to describe and understand the existing status of AMR in Africa, in relation to common causes of infections, and antibiotics recommended in treatment guidelines, an interesting review was published in 2017 using a public health focused approach [26]. The review identified three important findings: (1) for more than 40% of the countries, recent AMR data was not available; (2) the level of AMR to commonly prescribed antibiotics was significant; (3) the quality of microbiological data was of serious concern. According to the authors’ conclusions it was mandatory to address the gaps in AMR diagnostic standardization and reporting and use available information to improve and optimize treatment guidelines.

The drivers of AMR in Lebanon are multifactorial due to the absence of adequate national surveillance system, the lack of regulations controlling the agricultural industry, the low level of public awareness about antibiotics and the poor management of sanitation and waste. The concept of “healthy humans, animals and environment” is not part of any national plan nor is it a priority on the Lebanese government agenda. The concept of “One Health” is non-existent except for few efforts from non-governmental organization. In Lebanon, antibiotics are available over the counter [27], and self-medication is a common practice [28]. A study conducted in the capital region and its suburbs showed that around 40% of antibiotic therapy is prescribed by the community pharmacist [28]. No study to date has evaluated the impact of leftover antibiotics on resistance. Other patient factors are related to the inadequate duration of therapy, inappropriate indication for use, non-adherence to the antibiotic regimens, and lack of awareness about antibiotic uses [29]. In the hospital sector, it is found that, in addition to the prescriber factors listed above, drivers of resistance may be due to poor applicability of antibiotic stewardship programs, poor infection control and prevention practices and the selection pressure due to the misuse and abuse of disinfectants and decontaminants [30].

**Figure 1:**
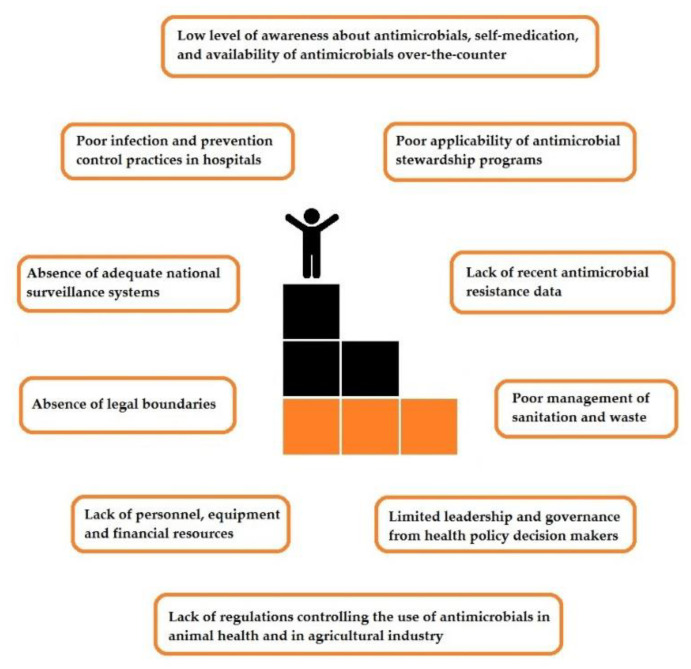
Challenges to be overcome in facing antimicrobial resistance in low- and middle-income countries.

Significant challenges in LMICs include the availability of antibiotics over-the-counter, the spread of counterfeit medications, uses of antibiotics in animal health and the agricultural sector in the absence of legal boundaries and limited leadership and governance from health policy decision makers. In an era of limited resources, LMICs are facing major hurdles that shift the focus away from this pandemic that is silently expanding in scope and in scale and reaching not only the individual but also the society, the nation and the entire global ecosystem. Challenges to be overcome in facing AMR in LMICs are reported in Figure 1.

Furthermore, addressing AMR in LMICs requires strong political will. Continuous human, infrastructural, and operational resources are needed in order to broadly recognize the nature and extent of AMR, and consequently to implement and update regional and national action plans for AMR prevention and containment within national health systems. Action plans require a multidisciplinary partnership among private and public sectors, civil society and non-profit organizations, patients, healthcare professionals, and communities, and multinational pharmaceutical industry and governments, supported by clear international, national, and regional policy frameworks that suspend private interests for public good [30].

In order to preserve the positive externality of antibiotics, that is the benefit of these medications for the future generations, mitigating AMR should start by fostering the correct use of antibiotics within the One Health framework such as restricting access to antibiotics and shifting status to prescription only for use in human health. The role of antibiotics in animal health and the environmental sector is a crucial part of the solution which must be better regulated but this is not in the scope of this review. In healthcare sectors, applying evidence-based infection control and prevention and implementing antibiotic stewardship programs adapted to the limited resources and expertise pitfalls. In this complex setting physicians are faced with major challenges related to inappropriate microbiology results, questionable infection control practice, limited human resources, lack of public awareness and knowledge about AMR. The good news is that there has been substantial progress in some LMICs, particularly in Africa and especially in South Africa, with the establishment of Antibiotic Surveillance and Stewardship programs in a number of private and public hospitals, with resultant changes in prescribing practices, including those of surgeons. Hospital-based pharmacist-led programs have been instrumental in improving basic hygiene practices and reducing sepsis through hand-washing campaigns, despite numerous challenges common in LMIC’s (staff shortages, internet connectivity issues, staff turnover, etc.) [31], and the implementation of group-wide infection-prevention in critically-ill patients, such as central-line sepsis prevention strategies and the “Best-care Always” [32] campaign. This was possible in Africa because of the “Ubuntu” concept: “I am who I am because of others” that engendered buy-in from the teams involved. This has led to reductions in SSIs as well.

## 4. Antibiotic Prescribing Practices across the Surgical Pathway in LMICs

A major proportion of the antibiotics prescribed within hospitals are for surgical patients. As observed in a recent worldwide cross-sectional survey, although most surveyed surgeons are aware of the problem of AMR, they underestimate this problem in their own hospital [33]. Both poor and inadequate infection and prevention control measures, and inappropriate use of antibiotics are contributing to the development of AMR.

Appropriate use of antibiotics is an important part of any antimicrobial stewardship program (ASP), and it is necessary to decrease the emergence and dissemination of AMR, and—as a consequence—to guarantee both good clinical practice, and optimal standards of care. Nevertheless, antibiotic prescribing practices among surgeons are often inappropriate, and across the surgical pathway, a noteworthy gap exists between the best evidence and clinical practice [19].

Surgical antibiotic prophylaxis (SAP) plays a pivotal role in a perioperative infection prevention approach [34,35,36]. The use of SAP remarkably contributes to the total amount of antibiotics used in hospitals and healthcare facilities, and may be correlated to increases in AMR and healthcare costs. SAP is one of the most important factors in reducing the rate of SSIs, but also consideration to basic infection control strategies may have a deep effect on SSIs rates. The WHO report on the burden of healthcare-associated infection illustrates that the incidence rate of SSI in LMICS range from 1.2–23.6% [37]. Different studies point to inappropriate SAP as an important cause for SSI [38], and some studies indicate that SAP is not delivered properly in LMCIs [39]. Programs to improve compliance with antibiotic prophylaxis protocols have shown good results when guided and monitored by hospital pharmacists and supportive surgeons [40]. This situation is worsened by the absence of data on the biology of SSIs to inspire guidelines and protocols specifically adapted to these settings.

Antibiotic therapy has an important role in the management of surgical infections, particularly in critically ill patients who need immediate empiric antibiotic therapy. Both poor antibiotic coverage, and inappropriate regimens represent the factors most strongly associated with unfavourable outcomes [41]. The dose, regimen, timing, route of administration, and duration of antibiotic therapy always have to be optimized. In most patients with surgical infections, after adequate source control, the aim of antibiotic therapy is to treat any residual infection. In these patients, the prolongation of antibiotic treatment beyond the duration suggested by established guidelines not only may lead to AMR, but also does not prevent the persistence or recurrence of the infection. Once an adequate source control is achieved, the duration of antibiotic therapy should be shortened as much as possible, unless specific clinical conditions requiring a prolongation of the antibiotic therapy occur (such as signs of an ongoing infection) [42].

**Figure 2:**
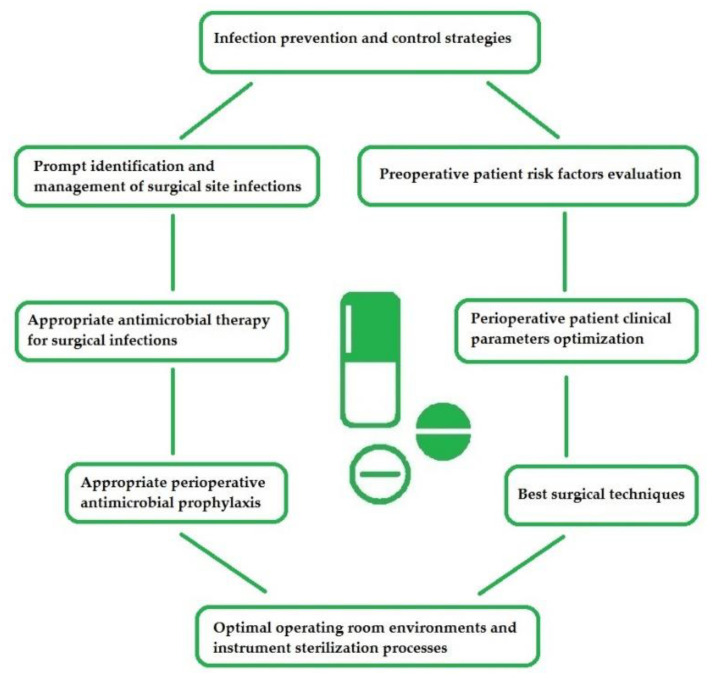
Antimicrobial stewardship: the surgeon’s role.

Surgeons play an essential role in prevention and treatment of infections (Figure 2), and their practices must be evidence-based. In most LMICs a high patient-doctor ratio exists, surgeons are overwhelmed and there is often inadequate time for meaningful education on adherence guidelines, leading to poor or non-adherence to these guidelines for the management of surgical infections. Antibiotic treatment is often the administration of broad-spectrum antibiotics without taking into account a definitive microbiological diagnosis, and indication for antibiotic treatment [43]. This can be improved with collaborative team approaches [14,44].

Because of the lack of reliable and effective surveillance systems, in LMICs all physicians and surgeons can lack updated information on the AMR pattern within their populations they take care of. In particular it occurs in rural settings where the absence of capacity to perform AMR testing imply difficulties to select the correct antibiotic in the absence of an antibiotic sensitivity test, and here guidance from National Antibiotic Guidelines can be useful as an adjunct for appropriate empiric antibiotic selection [45]. As a result, more and more broad-spectrum antibiotics are used to treat infections. This practice contributes to the development of resistance. Some of these resistance profiles are becoming untreatable with the current armament of antibiotics, which severely limits the options open to the surgeon or the intensivist treating these unfortunate patients [46].

While the World Health Organization has reviewed the list of available antimicrobials and classified some as “critically important antimicrobials” [47], assess to these drugs is highly variable in LMIC’s, where many of the older classes of drug are available, yet the newer classes may not be so available. As such, while a number of the recent studies from LMICs reveal extensive AMR, the presence of carbapenem resistance is less in LMICs, although it is an increasing challenge, especially in Southern Africa [46].

ASPs have been implemented to optimize antibiotic utilization, reduce the emergence and spread of AMR, and improve patient outcomes. Nonetheless, the best strategies for ASPs are not absolutely established, and are likely to be different based on local routine clinical practice, culture, and policy, and probably due to limited available resources in LMICs. Although ASPs are needed in LMICs, in these countries all the components necessary for their successful implementation are seldom in place. While in high income countries, hospital ASPs typically include a multidisciplinary committee, continuous monitoring of antibiotic use and their resistance patterns and evaluation of intervention outcomes, along with development of evidence based local treatment guidelines and antibiotic formularies. However, these components are either not present at all or exist at a bare minimum level due to the above-mentioned limitations of human and organizational resources, infrastructure, and funding in LMICs [48].

In order to evaluate the effectiveness of ASPs in hospitals in LMICs an interesting systematic review was published in 2018 [49]. The majority of the considered studies described a positive effect of antimicrobial stewardship interventions for hospitalized patients. Nevertheless, these research investigations were performed in tertiary care centers of urban areas in middle-income countries, which narrow the generalizability of the results. Wide differences were observed in terms of resources, organization, prescription practices, and financing processes among countries and among healthcare facilities within countries. Taking into consideration the available evidence, the authors concluded that general recommendations regarding the effectiveness of antimicrobial stewardship interventions in LMICs could not be established.

Programs to improve antibiotic prescribing practices among surgical patients in South Africa have shown promise. In both the private sector and the public sector institution of various programs have improved prescribing practises and ensured compliance with step-wise antibiotic protocols based on surveillance and antibiotic stewardship. These programs have shown reductions in unnecessary antibiotic use, redundant drugs and unnecessary prolonged treatment durations [44]. Even empiric antibiotics and treatment of post-operative complications is improved with good surveillance and appropriate antimicrobial stewardship, within the public hospital sector in South Africa, leading to a correct selection of antibiotic in over 93% of cases [50]. For this concept to work, enthusiastic surgeons, infectious diseases specialists, microbiologists, and pharmacists are required. Two quality indicators for future implementation have been recently recommended namely, tailoring empirical antibiotic therapy according to national antibiotic guidelines and assessing antibiotics prescribed against recommended national antibiotic guidelines [44].

## 5. Strategies for Prudent Antibiotic Prescribing Practices in LMICs

Surgical infections largely contribute to the massive problem of infectious diseases worldwide. One recent scoping review revealed that supply-side intervention strategies are the most successful approach to rectify irrational practices and promote prudent prescription of antibiotics; thereby, this tactic has been considered as the principal protector of overuse, misuse, irrational consumption of antibiotics in LMICs [51]. Another study recommended that LMICs should develop their own policy and planning in the contact of local culture and healthcare issues regarding antimicrobial stewardship strategies to promote appropriate prescribing practices. Strategic points might need to be progressively addressed in LMICs, such as: (1) the proper diagnostic facilities; (2) educational intervention for both health professionals and ordinary people; (3) national medicine regulatory agencies need to be strengthened with the support of well-established highly reputed agencies such as Food and Drug Administration (FDA), a federal agency of the USA, to audit the pharmaceutical industry and their marketing strategy, prescribing practice, pharmacy to control over the counter sale and dispensing of antibiotics; (4) overall improvement of healthcare facilities; (5) inter and intradepartmental cooperation between all stakeholders of the healthcare system; and (6) the need to formulate easily implementable antimicrobial stewardship for both hospital service and of community-level [52]. Another recent study reported that difficulties in the treatment strategies of surgical infections in LMICs include a scarcity of trained health professionals and infection-related resources constraints. This study further recommends for the development of antibiotic sensitivity surveillance programs, adequate infection prevention policy, planning, and implementation, and antimicrobial stewardship remains as the primary phase to look forward. Additionally, regular educational interventions among all healthcare professionals are of the utmost importance, and enlightening interference regarding AMR must begin before a medical student or other health professionals graduate, prior to housemanship, and continuing medical education (CME) throughout the working life. These teaching and learning sessions should adopt active learning methods and need completion as a part of a professional competence to maintain their medical registration or prescriber licenses [53].

## 6. Conclusions

Although the control of AMR cannot be the sole responsibility of healthcare professionals, they have a pivotal role in containing it. Continuing education is essential to enable physicians learning the need for rational prescription practices and the importance of evidence-based prescription. Antibiotic misuse and overuse is a problem observed worldwide, not only in high income countries, but in LMCIs as well. The requisite to formulate, communicate, and adopt rigorous policies for appropriate antibiotic use is more pressing in LMCIs where the greatest levels of abuse are encountered. This is not difficult but requires a compliant mindset change and teamwork, even in rural or low-resourced settings.

The surgeon’s role is paramount in preventing and managing infections which often need a prompt source control, and an appropriate antibiotic therapy, being directly responsible for their outcome. Surgeons must improve the quality of surgical care and avoid inappropriate antibiotic prescribing in surgery, with patients at risk of receiving prolonged durations of antibiotics for prophylactic and therapeutic indications.

A well-established antibiotic policy would go a long way towards achieving a reduction of inadequate prescribing. Moreover, the issue can be addressed with a reasonable degree of success when prescribing surgeons have adequate relevant knowledge of both the properties of antibiotic agents and the pathogens that are likely to cause infection or resistance profiles to commonly available antibiotics.
